# Comparative efficacy and safety of Sofosbuvir/Velpatasvir and Danoprevir for the treatment of chronic hepatitis C: the real-world data in China

**DOI:** 10.1186/s12876-024-03147-5

**Published:** 2024-02-14

**Authors:** Yunjing Zhou, Minfeng Liang, Yiting Li, Xing Chen, Jie Yang, Honglian Bai, Yingzi Long, Xiaohong Zhang, Chaoshuang Lin

**Affiliations:** 1 Department of Infectious Diseases, The Third Affiliated Hospital of Sun Yat-Sen University, No. 600 Tianhe Road , 510630 Guangzhou City, Guangdong Province, China; 2 Department of Infectious disease, The First people’s hospital of Foshan, Foshan City, Guangdong Province, China; 3 Department of General Practice, The Third Affiliated Hospital of Sun Yat-Sen University, Guangzhou City, Guangdong Province, China; 4 Department of Infectious Diseases, Yangjiang Public Health Hospital, Yangjiang City, Guangdong Province, China; 5 Clinical Medicine Department of Southwest Medical University, Luzhou City, Sichuan Province, China; 6 Department of Infectious Diseases, The Eighth Hospital of Guangzhou Medical University, Guangzhou City, Guangdong Province, China

**Keywords:** Sofosbuvir/Velpatasvir, Danoprevir, HCV genotype, Virological response, Adverse effects

## Abstract

**Background:**

Sofosbuvir/Velpatasvir (Epclusa, ECS) is the first pan-genotype direct-acting antiviral agent (DAA) for hepatitis C virus (HCV) infection, and Danoprevir (DNV) is the first DAA developed by a Chinese local enterprise, which is suitable for combined use with other drugs to treat genotype 1b chronic hepatitis C. However, previous reports have never compared the real-world data of ECS and DNV.

**Patients and methods:**

178 chronic hepatitis C patients were retrospectively recruited, and 94cases were accepted with Sofosbuvir/Velpatasvir ± Ribavirin (ECS group), and others (*n* = 84 treated with DNV combination therapy (DNV group). The HCV genotype, virological response, adverse effects and some laboratory biochemical indexes were contrasted between above two groups in the real world study.

**Results:**

DNV group had significantly lower level of alpha-fetoprotein (AFP), lower rates of decompensated cirrhosis ( *P* < 0.05). ECS group possessed more 6a (31.91% vs.13.10%) while DNV group was provided with more 1b (48.81% vs. 22.34%) patients. Significantly poor liver function was detected in ECS group at 4-week treatment (ALT and AST) and 12-week follow-up (AST) (all *P* < 0.05). The SVR12 undetectable rates of both groups were 100%, and no serious event was observed during the treatment and follow-up in both groups.

**Conclusion:**

In this retrospective real-world study, the efficacy of DNV combined therapy is similar to Sofosbuvir/Velpatasvir ± Ribavirin for chronic HCV infection, and the safety is comparable. DNV based therapy is a promising regimen for chronic hepatitis C.

## Introduction

Sofosbuvir/Velpatasvir (Epclusa, ECS), a fixed-dose combination tablet containing Sofosbuvir and Velpatasvir, has been approved by FDA in June, 2016 for the treatment of infection with hepatitis C virus (HCV) genotypes 1 through 6 [1]. It is the first to treat all six major forms of HCV [1, 2]. Moreover, ECS is also labeled for use in combination with the drug Ribavirin to treat patients with moderate to severe cirrhosis. It was listed in China in 2018. In June, 2018, the first, indigenous, class 1 innovative anti hepatitis C drug gonovir, Danoprevir (DNV) developed by Ascletis was approved by National Food and Drug Administration (NMPA) [3]. DNV is a potent macrocyclic inhibitor of the HCV NS3/4A serine protease. DNV is the first direct-acting antiviral agent (DAA) developed by a local enterprise in China. It is suitable for combined use with other drugs to treat newly diagnosed non cirrhotic genotype 1 chronic hepatitis C. Besides, both ECS and DNV belong to DAA, which have emerged as simple, short, safe, and effective treatments for chronic hepatitis C [4]. In the real world of China, a developing country, is characterized by unbalanced regional development. Clinical medication will be affected by drug accessibility, patient willingness, economic characteristics and other factors [5]. However, there is no report comparing real-world data of the two drugs listed in China in the same year. From July 2018 to December 2019, we conducted a real-world study in three cities with different economic levels in China, aiming to compare the efficacy and safety of Sofosbuvir/Velpatasvir and Danoprevir for chronic HCV infection.

## Patients and methods

### Patients collection and research design

Patients with chronic hepatitis C were retrospectively recruited from The Third Affiliated Hospital of Sun Yat-Sen University (Guangzhou, Guangdong, China), The First people’s hospital of Foshan (Foshan, Guangdong, China), Yangjiang Public Health Hospital (Yangjiang, Guangdong, China), The Eighth Hospital of Guangzhou Medical University (Guangzhou, Guangdong, China) and Clinical Medicine Department of Southwest Medical University (Luzhou, Sichuang, China). The criteria include: (1) age > 18 years old; (2) diagnosis as chronic hepatitis C (HCV genotype unlimited); (3) without DAA treatment history before enrollment. The exclusion criteria were: (1) liver transplantation patients; (2) pregnant women; (3) patients who are allergic to the study drug. The cohort included patients with diabetes, syphilis, and cancer (liver cancer). They were separated into 2 groups by treatment protocols, some accepted with Sofosbuvir/Velpatasvir ± Ribavirin (ECS group) for 12 weeks, and others treated with DNV combination therapy (DNV group) for 12 weeks. In the ECS group, patients took Sofosbuvir/Velpatasvir (Sofosbuvir 400 mg + Velpatasvir 100 mg tablets, Gilead Sciences, Inc., California, USA), one tablet orally once a day for 12 weeks, with or without food. If patients merged with decompensated cirrhosis, they accepted Sofosbuvir/Velpatasvir and Ribavirin (1000 mg/day for ≤ 75 kg patients, 1200 mg/day for > 75 kg patients) for 12 weeks. In the DNV group, patients treated with DNV (100 mg each time, twice a day) + Sofosbuvir (400 mg, once a day) ± Ribavirin (adjusted according to body weight). And some of patients trearted with DNV + Peginterferona IFα-2a Solution for Injection (Pegasys, 180 UG, once a week, subcutaneously injected into abdomen or thigh) ± Ribavirin. For patients with HIV infection, Ritonavir Tablets were added (600 mg orally, twice a day, preferably with food). Afterwards, all cases were followed up for 12 weeks. The primary endpoint was the rate of sustained virologic response all week 12 after the end of treatment (SVR12). The secondary endpoint was virologic response rate at end - of - treatment (EOT) and adverse event outcome. All patients who participated in this study signed an informed consent form, and this study was approved by institutional ethics board of China Ethics Committee of Registering Clinical Trials (No. ChiECRCT-20,190,013). All procedures performed in this study involving human participants were in accordance with the Declaration of Helsinki (as revised in 2013).

### Detection indexes and efficacy evaluation

Patient baseline demographic variables included age, sex, HCV genotype, body mass, body mass index (BMI), and comorbidities (including hyperlipidemia, hypertension, diabetes, and cancer). The levels of alpha-fetoprotein (AFP), alanine aminotransferase (ALT), aspartate aminotransferase (AST), total bilirubin (TBIL), direct bilirubin (DBIL) and HCV RNA were measured at baseline, 4, 8, 12 and 24 weeks of treatment. According to manufacture process, HCV RNA was tested with COBAS * AmpliPrep / COBAS * TaqMan * HCV Quantitative Test Kit ( Life Technologies, USA) with the lower limit of detection 15 IU / ml. The patient underwent abdominal B-ultrasound at the initial diagnosis to determine whether it was liver cirrhosis. The safety evaluation of the treatment plan includes the types and quantities of adverse events and serious adverse events observed during treatment and follow-up.

### Statistical analysis

Continuous data were indicated with mean ± standard deviation (SD) while categorical data were indicated with number and percentage (%). For comparisons of means between groups, Mann-Whitney U test was used. Categorical data were tested using Chi-square test or Fisher’s exact text (if expected value ≤ 5 was found). Line chart was used to illustrate the changing trend of ALT and AST among time-points, including baseline, 4-week treatment, 12-week treatment (at the end of treatment), and 12-week follow-up (SVR 12). The HCV RNA undetectable rates were also reported among HCV genotypes. A *P* < 0.05 would be recognized as reaching significance of each test, two-tailed. All above analyses were performed using IBM SPSS Version 25 (SPSS Statistics V25, IBM Corporation, Somers, New York).

## Results

### Patient’s clinical characteristics

A total of 178 HCV infected patients were included in this study with the medium age of 48 (22–82) years old, and the gender ratio was 1.83:1 (male/female = 115/63/). 19 (10.61%) of patients had different comorbidities, such as cirrhosis, diabetes, hypertension, liver cancer. The averaging HCV RNA level was 5.73 ± 1.10 log10 (IU/mL). These patients were separated into 2 groups by treatment protocols: 94 in ECS group and 84 in DNV group. The percentage of HBV co-infection is 3.19% in ECS group and 2.38% in DNV group. There are 3 patients co-infected with HBV in ECS group and 2 patients in DNV group. Besides, the percentage of HIV co-infection is 1.06% in ECS group and 0.0％ in DNV groups. ECS group has one patient co-infected with HIV. And DNV group has no patients co-infected with HIV.

Table 1 demonstrates all clinical characteristics between protocol groups. As indicated, it seemed that patients in DNV group had significantly lower level of alpha-fetoprotein (AFP), lower rates of cirrhosis and decompensated cirrhosis (all *P* < 0.05). The HCV genotype also differed between ECS and DNV groups, ECS group possessed more 6a (31.91% vs. 13.10%) while DNV group was provided with more 1b (48.81% vs. 22.34%) patients.

**Table 1:** Clinical characteristics between patients with different treatment protocols

Parameters	ECS (*n* = 94)	DNV (*n* = 84)	All (*n* = 178)	*P*
Age, year	48.88 ± 10.66	47.60 ± 10.32	48.27 ± 10.49	0.417
Sex				0.098
Male	66 (70.21%)	49 (58.33%)	115 (64.6%)	
Female	28 (29.79%)	35(41.67%)	63 (35.4%)	
Comorbidity				0.054
No	80 (85.11%)	79 (94.05%)	159 (88.83%)	
Yes	14 (14.89%)	5 (5.95%)	19 (10.61%)	
Log_10_(HCVRNA), log_10_(IU/mL)	5.73 ± 1.10	6.03 ± 0.96	5.89 ± 1.04	0.408
AFP, ng/mL	42.84 ± 93.906	5.91 ± 5.28	21.16 ± 62.79	0.012
HCV genotype				< 0.001
1a	2(2.13%)	1 (1.19%)	3 (1.69%)	
1b	21 (22.34%)	41 (48.81%)	62 (34.83%)	
2a	7 (7.45%)	8 (9.52%)	15 (8.43%)	
3a	7 (7.45%)	10 (11.90%)	17 (9.55%)	
3b	12 (12.77%)	10 (11.90%)	22 (12.36%)	
6a	30 (31.91%)	11 (13.10%)	41 (23.03%)	
6e	1(1.06%)	0(0.00%)	1(0.56%)	
NA	14(14.89%)	3(3.57%)	17(9.55%)	
Cirrhosis				0.054
No	80(85.11%)	79 (94.05%)	159 (89.33%)	
Yes	14 (14.89%)	5(5.95%)	19 (10.67%)	
Decompensated cirrhosis				0.006
No	86 (91.49%)	84 (96.97%)	170 (95.51%)	
Yes	8 (8.51%)	0 (0)	8 (4.49%)	

### Laboratory results among time-points

Laboratory indexes were statistically analyzed, including ALT, AST, WBC, Hb, and PLT, and results were exhibited in Table 2. As indicated in Table 2, AST and ALT levels rapidly dropped after the starting of treatment (both *P* < 0.001). Significantly poor liver function was observed in ECS group at 4-week treatment (ALT and AST) and 12-week follow-up (AST) (all *P* < 0.05).

**Table 2:** Laboratory results between treatment protocol groups from baseline to follow-up

Parameters	ECS (*n* = 94)	DNV (*n* = 84)	All (*n* = 178)	*P*
Baseline				
ALT^a^, U/L	82.54 ± 75.39	73.48 ± 54.65	78.04 ± 65.82	0.406
AST^b^, U/L	66.57 ± 45.63	60.44 ± 42.54	63.53 ± 44.08	0.401
WBC^c^	6.37 ± 1.60	6.35 ± 1.87	6.16 ± 1.96	0.177
Hb^d^	144.28 ± 20.49	144.28 ± 20.49	137.40 ± 29.72	0.101
PLT^e^	154.06 ± 75.00	176.78 ± 66.93	165.01 ± 71.88	0.062
4-week treatment				
ALT, U/L	30.51 ± 20.25	22.28 ± 10.51	26.28 ± 16.47	0.007
AST, U/L	33.13 ± 14.78	23.58 ± 7.63	28.23 ± 12.56	< 0.001
WBC	5.65 ± 2.11	6.10 ± 1.22	5.77 ± 1.92	0.416
Hb	134.53 ± 21.09	137.08 ± 17.19	135.62 ± 19.45	0.544
PLT	161.82 ± 73.85	173.00 ± 68.94	166.60 ± 71.61	0.470
12-week treatment				
ALT, U/L	27.29 ± 21.76	23.58 ± 10.28	26.49 ± 18.32	0.209
AST, U/L	31.92 ± 20.39	24.31 ± 8.82	29.01 ± 17.27	0.030
WBC	6.02 ± 1.81	5.58 ± 1.77	5.86 ± 1.80	0.271
Hb	136.38 ± 24.63	136.00 ± 18.69	136.33 ± 23.92	0.969
PLT	162.23 ± 73.57	199.75 ± 62.68	175.87 ± 71.77	0.029
12-week follow-up				
ALT, U/L	26.38 ± 7.38	17.24 ± 3.90	23.88 ± 7.76	< 0.001
AST, U/L	26.38 ± 7.38	17.24 ± 3.90	23.88 ± 7.76	< 0.001
WBC	6.32 ± 2.12	6.58 ± 0.983	6.36 ± 1.80	0.748
Hb	140.59 ± 19.19	136.00 ± 18.69	139.86 ± 18.97	0.563
PLT	171.54 ± 57.25	252.14 ± 56.84	184.36 ± 63.91	0.001

^a^ ALT’s reference value is 3–35 U/L

^b^ AST’s reference value is 13–35 U/L

^c^ WBC’s reference value is 4.1–11.0 × 10^9/L

^d^ Hb’s reference value is 114–154 g/L

^e^ PLT’s reference value is 100–350 × 10^9/L

### HCV RNA undetectable rates and adverse effects

Table 3 showed the HCV RNA undetectable rates and adverse effects of patients in both groups among time-points. As indicated, the undetectable rates rapidly increasing after the starting of treatment, reached 88.42% at week 4 and 100% at 12-week follow-up (SVR12) in both groups. In detail, the 4-week treatment, 12-week treatment (at the end of treatment) and SVR12 undetectable rates of ECS group were 88.42%, 100% and 100%, respectively. In the DNV group, they were separately 72.62%, 97.62% and 100%. Notably, significant higher rate was displayed in ECS group (88.42% vs. 72.62%) at 4-week treatment (*P* = 0.004).

**Table 3:** The HCV RNA undetectable rates and adverse effects among time-points

Parameters	ECS(*n* = 94)	DNV (*n* = 84)	All (*n* = 178)	*P*
HCV RNA undetectable				
4-week treatment	84 (88.42%)	61 (72.62%)	145 (81.46%)	0.004
At the end of treatment	94(100.00%)	82 (97.62%)	176 (94.25%)	0.132
SVR12	94(100.00%)	84(100.00%)	178 (100.00%)	1.000
Adverse effects				0.331
No	92 (97.87%)	80 (95.24%)	172 (96.63%)	
Yes	2 (2.13%)	4 (4.76%)	6 (3.37%)	

SVR12, Undetectable HCV RNA 12 weeks after the end of treatment

In all enrolls, 6 (3.37%) cases occurred adverse effects. Further, no significant difference was found in the adverse effect rates between groups (2.13% vs. 4.76%, *P* = 0.331). In ECS group, 2 patients had the problem of poor sleep; in DNV group, 1 patient with increasing heart rate, 1 patient with headache, 1 patient with decreasing WBC level, and 1 patient with increasing total bile acids. No new adverse events occurred, and all of them were controllable.

## Discussion

From the patient’s clinical baseline, it suggested that patients with higher AFP level and comorbidity tended to accepted ECS. Patients with higher AFP (> 20ng/ml) possess higher hepatocellular carcinoma (HCC) incidence rate and worse prognosis, one of the most malignant cancer with high mortality [6]. Moreover, AFP plays a key role in stimulating the growth, metastasis and drug resistance of HCC, and has been widely used for screening and monitoring HCC [7, 8]. In this cohort, 8 patients with HCC were included, and all of them chose ECS. In the real world, it is considered that patients with more complicated conditions are more inclined to use imported drugs (ECS) to control diseases. This conclusion is also supported by the higher proportion of cirrhosis and decompensated cirrhosis in ECS group (cirrhosis: 14.88% vs. 5.95%, *P* = 0.054; decompensated cirrhosis: 8.51% vs. 0, *P* = 0.006). Besides, these patients have higher clinical compliance, follow the doctor’s advice, timely reexamine, and actively receive follow-up. As for the HCV genotype, DNV group was provided with more 1b, because of the approved indication of DNV. Although NMPA only approved DNV for type 1b, in our real-world study, about 50% other genotypes were involved, and the SVR12 undetectable rates of Genotypes 1, 2, 3and 6 in DNV group were 100%. The reason lied in that most of them were accepted with DNV + Sofosbuvir ± Ribavirin.

Sofosbuvir is a pan-genotypic nucleoside NS5B polymerase inhibitor, which can effectively inhibit the replication of HCV genotypes 1–6 [9]. Sofosbuvir + Ribavirin ± Peginterferona IFα-2a, as well as Sofosbuvir + Ledipasvir or Velpatasvir (both of them are NS5B polymerase inhibitors), has been approved for the treatment of patients with genotype 1–6 chronic hepatitis C in China [10]. The feasibility has been explored by previous study that the DNV plus Sofosbuvir ± Ribavirin treated for HCV genotype 1, 2, 3, or 6 in 58 chronic hepatitis C patients with or without cirrhosis in China [11]. Based on these theories and studies, our results yielded an encouraging data with 100% SVR12. Even more, the results of this study show that both the DNV group’s and ECS group’s treatments in the genotype 3 chronic HCV-infected patients have a high efficacy and barrier to resistance, and significantly improves the inhibition of viral replication for the purpose of antiviral therapy. It indicated that DNV plus Sofosbuvir + Ribavirin could be an effective regimen for the treatment of multigenotype chronic hepatitis C. The proportion of genotype 3a in ECS group is 7.45% and 11.90% in the DNV group. Besides, in the ECS group, the proportion of genotype 3b is 12.77% and 11.90% in the DNV group. The SVR12 rates of these patients are encouragingly 100%. In China, HCV genotype 1b is the main prevalent type. However, in recent years, the prevalence of chronic hepatitis C genotype 3 infection in China is second only to type 1b and type 2a in the southern and southwestern regions with a prevalence rate of 13.69% [12]. Compared with other genotypes, genotype 3 chronic HCV-infected patients have more rapid progression of liver fibrosis and steatosis and an increased risk of developing hepatocellular carcinoma. The results of this study show that in type 3 chronic HCV-infected patients both ECS and DNV also have high efficacy and barrier to resistance, and significantly improves the inhibition of viral replication for the purpose of antiviral therapy. The patients enrolled in this study came from Guangdong provinces, and in this region, the high proportion of patients with subtype 6a infection is a unique feature [13, 14]. Even more, 6a has become a local endemic in Guangzhou [12]. Our study further revealed the treatment characteristics of such patients in this area, and most of them chose ECS (Table 1).

The efficacy and pharmacokinetics of the two drugs were the most important points in our research. Approval of ECS was based on the results of four randomized trials (ASTRAL-1, -2, -3 and − 4) in treatment-naive and -experienced patients with HCV infection. ASTRAL-1 enrolled patients with or without cirrhosis, but excluded those with decompensated cirrhosis. Genotypes 1, 2, 4, 5 and 6 was involved in ASTRAL-1, and the SVR12 rate was 99% [15, 16]. ASTRAL-4 included patients with decompensated (Child-Pugh B) Cirrhosis, and all genotypes were contained. In the ASTRAL-4, the SVR12 rate of ECS plus Ribavirin was 94%, and it was only 83% of ECS alone [15, 17]. In the real world study, our medication plan fully referred to the ASTRALs trails and approved indications. Patients merged with decompensated cirrhosis received ECS + Ribavirin. If not, ECS single drug. More excitingly, the SVR12 rate was 100% (Table 3), which confirmed the excellent performance of ECS again. It has been reported the SVR12 of previous report [11] was also 100%, same to our report. Moreover, we recruited more patients, and firstly compared DNV combined therapy with the globally recognized drug (ECS). It was worth noting that patients in ECS group seemed to respond faster to drugs. The response rate was 88.42% at 4 weeks treatment and 100.00% at the end of treatment (12 weeks treatment). Nevertheless, in the DNV group, although the SVR12 rate of Genotypes 1, 2, 3 and 6 was also 100%, the response rate at 4 and 12 week treatments were not as amazing as the former (Table 3). Besides, poor liver function was observed in ECS group at 4-week treatment (ALT and AST) and 12-week follow-up (AST) (all *P* < 0.05). The trend of liver function improvement of the two groups was similar (Table 2).

Adverse events were another important aspect in clinical practice. In this research no serious event was observed during the treatment and follow-up in both groups. The incidence of adverse events was very low, and there was no crossover between the two groups.

We have to admit that this study has some limitations. Firstly, as a real-world study, the sample size is not large enough. The sample size of patients with certain characteristics is insufficient, which may affect the statistical results. For example, there are few patients with type 1a, 2a, 3a, 3b and 6e. Although this situation can truly reflect the regional characteristics, the insufficient sample size may mislead the comparison results and curative effects between the two groups. For another example, the adverse events of the two groups discussed above did not have any same symptoms. However, the number of patients with adverse events in the two groups is very small (2 cases in ECS group and 4 cases in DNV group), and it may be different after expanding the sample. Secondly, in the real world, these patients indeed not use drugs strictly according to the approved indications. Thirdly, as described previous, the baseline of the two groups of patients is inconsistent. The baseline of the ECS group is more serious and the condition is more complex. This different baseline is likely to lead to differences in efficacy or incompatibility between the two groups. Despite these shortcomings, the significance of our research could not be ignored. For the first time, our study truly reflected and scientifically compared the clinical characteristics, drug efficacy and toxic and side effects of ECS and DNV in Chinese chronic hepatitis C patients. More importantly, this study tried to use DNV for typing other than 1b, and the effect was very good. Last, but certainly not least, the efficacy of DNV combined therapy is similar to Sofosbuvir/Velpatasvir ± Ribavirin for chronic HCV infection, and the safety is comparable. DNV based therapy is an optional and promising scheme in clinical practice.

## Data Availability

The data and study materials that support the findings of this study will be available to other researchers from the corresponding authors on reasonable request.

## Abbreviations

ECS: Sofosbuvir/Velpatasvir(Epclusa)
DAA: Direct-acting antiviral agent
HCV: Hepatitis C virus
DNV: Danoprevir
ECS group: Sofosbuvir/Velpatasvir(Epclusa) ± Ribavirin
DNV group: DNV combination therapy
AFP: Alpha-fetoprotein
SVR: Sustained virological response
EOT: End - of– treatment
BMI: Body mass index
AST: Aspartate aminotransferase
ALT: Alanine aminotransferase
TBIL: Total bilirubin
DBIL: Direct bilirubin
SD: Standard deviation
HCC: Hepatocellular carcinoma
WBC: White blood cell
PLT: Platelets
ALB: Albumin
TB: Total bilirubin
